# Bilateral waveform analysis of gait biomechanics presurgery to 12 months following ACL reconstruction compared to controls

**DOI:** 10.1002/jor.26001

**Published:** 2024-12-04

**Authors:** Christin Büttner, Caroline Lisee, Elizabeth Bjornsen, Ashley Buck, Natália Favoreto, Alexander Creighton, Ganesh Kamath, Jeffrey Spang, Jason R. Franz, Troy Blackburn, Brian Pietrosimone

**Affiliations:** ^1^ Department of Exercise and Sport Science University of North Carolina Chapel Hill North Carolina USA; ^2^ Institute of Human Movement Science and Health Chemnitz University of Technology Chemnitz Germany; ^3^ Department of Kinesiology University of Georgia Athens Georgia USA; ^4^ Joint Department of Biomedical Engineering University of North Carolina at Chapel Hill and North Carolina State University Chapel Hill North Carolina USA; ^5^ Thurston Arthritis Research Center, School of Medicine University of North Carolina Chapel Hill North Carolina USA; ^6^ Deparment of Orthopaedics University of North Carolina Chapel Hill North Carolina USA

**Keywords:** functional mixed effects model, joint loading, knee osteoarthritis

## Abstract

The purpose of this study was to compare gait biomechanics between limbs and to matched uninjured controls (i.e., sex, age, and body mass index) preoperatively and at 2, 4, 6, and 12 months following primary unilateral anterior cruciate ligament reconstruction (ACLR). Functional mixed effects models were used to identify differences in gait biomechanics throughout the stance phase between the a) ACLR limb and uninvolved limb, b) ACLR limb and controls, and c) uninvolved limb and controls. Compared with the uninvolved limb, the ACLR limb demonstrated lesser knee extension moment (KEM; within 8–37% range of stance) during early stance as well as lesser knee flexion moment (KFM; 45–84%) and greater knee flexion angle (KFA; 43–90%) during mid‐ to late stance at all timepoints. Compared with controls, the ACLR limb demonstrated lesser vertical ground reaction force (vGRF; 5–26%), lesser KEM (7–47%), and lesser knee adduction moment (KAM; 12–35%) during early stance as well as greater vGRF (39–63%) and greater KFA (34–95%) during mid‐ to late stance at all timepoints. Compared with controls, the uninvolved limb demonstrated lesser KFA (1–56%) and lesser KEM (12–54%) during early to mid‐stance at all timepoints. While gait becomes more symmetrical over the first 12 months post‐ACLR, the ACLR and uninvolved limbs both demonstrate persistent aberrant gait biomechanics compared to controls. Biomechanical waveforms throughout stance can be generally described as less dynamic following ACL injury and ACLR compared with uninjured controls.

## Clinical Significance

Individuals with ACLR demonstrate less dynamic gait biomechanics waveforms bilaterally from the preop timepoint up to 12 months post‐ACLR, which may contribute to knee joint tissue breakdown. Further, gait retraining interventions need to consider bilateral approaches to normalize gait following unilateral ACLR.

## 1 INTRODUCTION

Individuals who sustain an anterior cruciate ligament (ACL) injury are at high risk for developing knee osteoarthritis (KOA), with a third of individuals developing KOA in the first decade following injury despite undergoing surgical ACL reconstruction (ACLR) and therapeutic rehabilitation.^1^ Aberrant gait biomechanical outcomes, including reduced surgical limb vertical ground reaction force (vGRF), knee flexion angle (KFA), knee extension moment (KEM), and knee adduction moment (KAM) have been associated with deleterious joint tissue metabolism,^2^, ^3^ altered tibiofemoral cartilage composition,^4^, ^5^, ^6^, ^7^, ^8^, ^9^ radiographic joint changes,^10^ and worse knee‐related symptoms in patients with ACLR.^11^, ^12^ Therefore, it is important to clearly characterize how gait biomechanics are affected by ACL injury at early post‐injury and post‐ACLR timepoints when patients are typically engaged in supervised ACLR rehabilitation^13^, ^14^ and early KOA‐related joint tissue changes have started to develop.^5^, ^8^, ^9^

Previous systematic reviews and meta‐analyses have highlighted key knowledge gaps negatively impacting the ability to comprehensively characterize aberrant gait biomechanics at early timepoints following ACLR.^15^, ^16^, ^17^ Specifically, previous studies lack early longitudinal data between preoperative timepoints (preop; i.e., following ACL injury but before ACLR) and 12 months post‐ACLR that may be most relevant for guiding supervised rehabilitation of aberrant gait biomechanics before returning to unrestricted physical activity. Further, most studies do not systematically compare the biomechanical changes exhibited in the ACLR limb to both the contralateral, uninjured limb and matched‐uninjured controls. Research indicates that the uninjured limb undergoes biomechanical and biological KOA‐related changes following primary, unilateral ACLR,^18^, ^19^, ^20^, ^21^, ^22^ suggesting that a comprehensive multi‐control analysis (i.e., contralateral limb and matched uninjured controls) is needed to understand the bilateral biomechanical effects of unilateral ACL injury. Finally, previous studies primarily focused on changes in discrete features of gait, such as peak magnitudes (e.g., peak vGRF, peak KFA). However, recent research has demonstrated that gait biomechanics of the ACLR limb can differ at multiple portions of stance compared to the uninvolved limb and controls,^5^, ^6^, ^19^, ^23^ and differences at portions of stance other than the peak magnitudes are linked to important changes in knee cartilage composition.^6^ Therefore, utilizing waveform analyses to evaluate the entire stance phase instead of just discrete points may provide greater insights into how gait differs between groups. Overall, it is important to improve our knowledge of the bilateral effects of ACL injury and ACLR on gait throughout the entire stance phase at early time points, especially since gait biomechanics can be modified with various traditional modalities (e.g., insoles, knee braces)^24^, ^25^ and emerging precision treatment strategies (e.g., real‐time gait biofeedback),^26^, ^27^ which have not yet been broadly adopted in clinical settings. Therefore, the results of this study are critical for the development of precision gait retraining interventions to normalize gait biomechanics and mitigate future KOA development.

This study aimed to overcome these specific limitations by comparing key gait biomechanics (i.e., vGRF, KFA, KEM, KAM) throughout the stance phase of gait between the a) ACLR and uninvolved limbs, b) ACLR and matched control limbs, and c) uninvolved and matched control limbs at preop, as well as 2, 4, 6, and 12 months post‐ACLR follow‐up timepoints. We hypothesized that gait biomechanics of the ACLR and uninvolved limbs would become more symmetrical over time, however, both limbs would demonstrate less dynamic and more sustained waveforms compared with controls at all timepoints, with the ACLR limb demonstrating larger differences from controls than the uninvolved limb.

## 2 METHODS

### 2.1 Study design

We conducted a prospective, longitudinal cohort study with nested case‐control comparisons of gait biomechanics (i.e., vGRF, KFA, KEM, and KAM) in individuals with primary unilateral ACL injury and uninjured matched controls. Gait biomechanics were collected at habitual walking speed during preop and at 2, 4, 6, and 12 months post‐ACLR in individuals with ACL injury and during a single data collection session for controls. Uninjured controls were matched based on sex (female/male), baseline age (±2 years), and baseline body mass index (BMI ± 4 kg/m^2^). The decision to use the right or left limb as the reference limb in each control participant was based on the limb that was injured on their matched ACLR counterpart. All study procedures were approved by the Institutional Review Board at the University of North Carolina at Chapel Hill and all participants provided written consent or assent and permission from their parent or legal guardian to participate in the study.

### 2.2 Participants

We recruited individuals with a primary unilateral ACL injury between the ages of 16 and 35 years who were planning to undergo ACLR from a single academic health system‐based orthopedic practice. We included individuals who underwent either primary unilateral arthroscopic bone‐patellar tendon‐bone, quadriceps, or hamstring autograft ACLR. We excluded individuals receiving an allograft, requiring ACLR revision surgery, or multiple ligament surgery, or individuals who sustained a lower extremity fracture during ACL injury, or were diagnosed with OA or any other disease that affects the knee joint, or were pregnant at the time of enrollment or were planning to become pregnant during the study period. All ACLR participants received supervised rehabilitation for at least 6 months following surgery.

**Figure 1:**
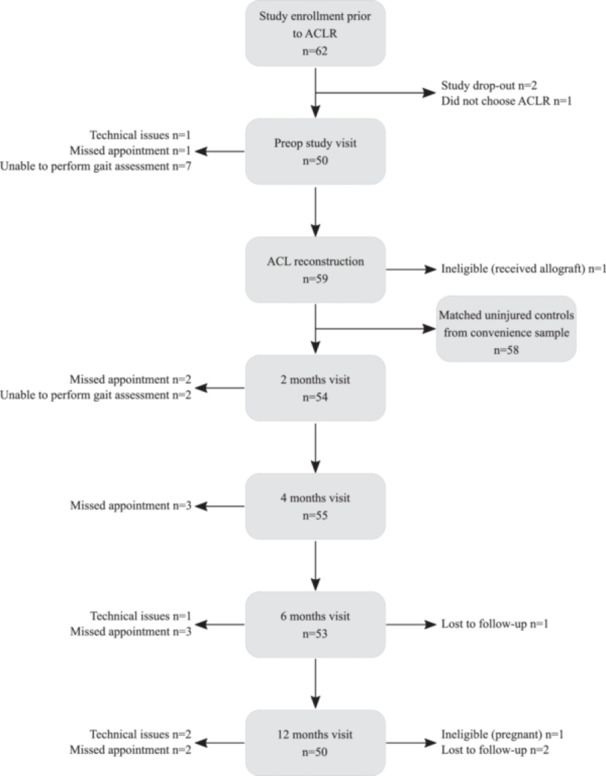
Strengthening the reporting of observational studies in epidemiology (STROBE) study flowchart.^28^ The reasons for missing gait data included: study dropout, lost to follow‐up, exhibiting an exclusion criterion following enrollment (e.g., chose not to undergo ACLR or became pregnant), missed appointments, inability to perform gait assessment, and technical data collection issues.

We enrolled uninjured control participants to match each ACLR participant for whom gait data was available for at least one timepoint (Figure 1). Controls were included if they had no history of any lower extremity orthopaedic surgery, no history of knee injury, no lower extremity joint injury or concussion within the last 6 months, no diagnosis of inflammatory arthritis, were not pregnant or had any other medical condition that could influence their gait or physical activity participation.

### 2.3 Biomechanical gait assessment

Gait biomechanics were collected as described in previous work^29^, ^30^, ^31^ using a 10‐camera 3D motion capture system (Vicon Bonita; Vicon Motion Systems Ltd, Oxford, UK) and three staggered and embedded force plates (FP406010; Bertec Corporation, Columbus, OH, USA) in a 6 m walkway. Participants were equipped with 29 retroreflective markers on the lower limbs, pelvis, and trunk and walked unshod and at their self‐selected habitual walking speed. Marker trajectories and kinetics were captured at 120 and 1200 Hz, respectively. We determined habitual gait speed from a minimum of 5 test trials using two timing gates (Dashr, Dashr, Lincoln, NE, USA) placed 0.97 m apart. Next, 5 successful trials of gait biomechanics were collected from both limbs at the habitual walking speed. A gait trial was considered successful if participants walked within ±5% of their average habitual speed of the test trials and struck the force plate with their entire foot (i.e. heal strike to toe off) during stance. Data were processed using custom scripts in Visual3D (version 2020) and Matlab (version R2022b) and filtered using a recursive fourth‐order low‐pass Butterworth filter with a cut‐off frequency of 10 Hz. Biomechanical outcomes were time‐normalized to 101 data points (0–100%) throughout the stance phase, which was defined as heel strike (vGRF>20 N) to toe‐off (vGRF<20 N). KFA was calculated as the angle of the shank relative to the thigh via Euler angles (sagittal/frontal/transverse sequence) and an inverse dynamics approach was used to calculate net internal knee moments. KEM and KAM were normalized to the product of bodyweight and height, and vGRF was normalized to bodyweight. Ensemble average waveforms were calculated for each outcome from the 5 recorded trials for statistical analysis.

### 2.4 Statistical analysis

#### 2.4.1 Descriptive data

Gait biomechanical data were available for at least one timepoint for a total of 58 out of 62 recruited participants with ACL injury (Figure 1). As a result, we analyzed datasets for the following number of participants at each timepoint in the ACLR group: preop n = 50; 2 months: n = 54; 4 months: n = 55; 6 months: n = 53; 12 months: n = 50 (Supplement 1; Table S1.1). For further analyses, all available gait data of the ACLR group were compared with the 58 matched control limbs at each timepoint.

Group differences between the ACLR and control group in age and BMI were assessed at preop and for gait speed at all timepoints using independent *t*‐tests or Mann‐Whitney‐U‐tests (α = 0.05) based on normality of the data. Furthermore, distribution of graft type, percentage of medial meniscal injury, lateral meniscal injury, chondral injury, and Knee Injury and Osteoarthritis Outcome Score (KOOS) outcomes for the ACLR group are reported in Table 1 and Table 2.

**Table 1:** Anthropometrics of the ACLR and control groups.

Characteristics	Timepoint	ACLR	Controls	p‐value
Sex (% females)		57.0	57.0	
Age (y)		21.8 ± 4.7	21.6 ± 4.3	0.95
BMI (kg/m^2^)		24.2 ± 3.2	24.5 ± 3.2	0.57
Gait speed (m/s)	control visit		1.36 ± 0.14	
	preop	1.16 ± 0.18		<0.001*
	2 months	1.17 ± 0.14		<0.001*
	4 months	1.24 ± 0.13		<0.001*
	6 months	1.25 ± 0.13		<0.001*
	12 months	1.27 ± 0.13		<0.001*
Days before ACLR	preop	10.6 ± 10.2		
Days since ACLR	2 months	56.6 ± 5.5		
	4 months	114.4 ± 6.3		
	6 months	174.6 ± 9.3		
	12 months	346.9 ± 15.8		
Graft type (PT/QT/H)		54/3/1		
Medial meniscal injury (%)		24.1		
Lateral meniscal injury (%)		69.0		
Chondral injury (%)		27.6		

* significant difference from Controls.

Abbreviations: ACLR, anterior cruciate ligament reconstruction; BMI, body mass index; H, hamstring; preop, preoperative; PT, patellar tendon; QT, quadriceps tendon.

**Table 2:** KOOS outcomes for the ACLR group at preop, 2, 4, 6, and 12 months post‐ACLR.

KOOS subscore	Timepoint	ACLR
Quality of life	preop	38.2 ± 18.7
	2 months	40.0 ± 13.6
	4 months	51.4 ± 17.2
	6 months	55.7 ± 17.0
	12 months	74.0 ± 20.9
Pain	preop	73.6 ± 12.3
	2 months	76.4 ± 11.5
	4 months	82.9 ± 9.8
	6 months	85.6 ± 9.1
	12 months	92.5 ± 7.6
Symptoms	preop	68.1 ± 14.4
	2 months	65.0 ± 13.1
	4 months	74.3 ± 14.5
	6 months	79.7 ± 11.6
	12 months	85.7 ± 10.9
Activities of daily living	preop	83.1 ± 13.9
	2 months	87.5 ± 9.0
	4 months	94.1 ± 7.3
	6 months	96.1 ± 5.7
	12 months	97.2 ± 5.0
Sport	preop	40.2 ± 22.0
	2 months	32.8 ± 23.9
	4 months	51.9 ± 20.1
	6 months	66.8 ± 18.6
	12 months	84.2 ± 19.8

Abbreviations: ACLR, anterior cruciate ligament reconstruction; KOOS, Knee Injury and Osteoarthritis Outcome Score; preop, preoperative.

#### 2.4.2 Gait biomechanical analysis

We conducted functional waveform analyses to evaluate differences between biomechanical outcomes throughout stance as previously described.^29^, ^30^, ^32^ Separate functional mixed effects models were compiled at each timepoint for gait biomechanical outcomes (i.e., vGRF, KFA, KEM, KAM) to compare differences between the (a) ACLR and uninvolved limbs, (b) ACLR limb and control limb, and (c) uninvolved limb and control limb. All participant's average gait waveforms from 5 successful trials were fit with Bayesian functional models using a B‐spline model to gain representative waveforms of the ACLR limb, uninvolved limb, and control group limb.^33^ The functional mixed effects models also computed the mean differences between the representative waveform of each group as well as their corresponding 95% confidence intervals and Cohen's *d* effect sizes. Statistically significant differences between waveforms were defined as areas in which the 95% confidence interval of the mean difference did not include zero. The waveform analyses were performed in RStudio (version 4.1.2) utilizing the FunctionalMixedEffects package.^33^ Since gait speed differed at all timepoints between the ACLR and uninjured controls and is linked to changes in biomechanical outcomes^34^ and KOA development following ACLR,^35^, ^36^ we also conducted the same waveform analysis as described while statistically controlling for the average habitual walking speed of each participant at each timepoint (i.e., preop, 2, 4, 6, and 12 months) relative to the average walking speed of all participants at the same timepoint.^29^, ^32^ As with the analyses without adjusting for gait speed, separate functional mixed effects models were conducted for all key outcomes (i.e., vGRF, KFA, KEM, KAM) at all timepoints between groups.

## 3 RESULTS

### 3.1 Descriptive outcomes

The ACLR group walked at significantly slower speeds at all 5 timepoints (*p *< 0.001) compared with controls. No differences in age (*p *= 0.95) or BMI (*p *= 0.57) were found between groups (Table 1).

### 3.2 Differences in gait biomechanical outcomes without gait speed adjustment

#### 3.2.1 Vertical ground reaction force

##### ACLR limb versus uninvolved limb

**Figure 2:**
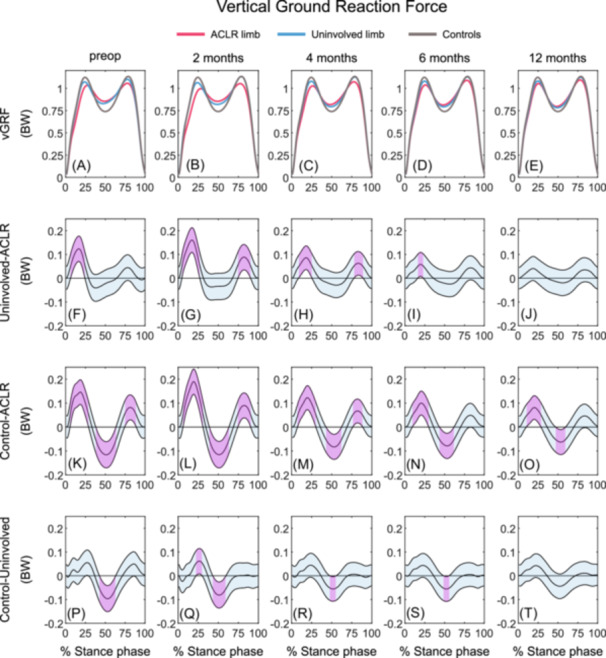
Mean vGRF waveforms and mean vGRF differences with 95% confidence intervals (light blue areas) throughout gait stance in the ACLR limb, uninvolved limb, and uninjured control limb at preop, 2, 4, 6, and 12 months post‐ACLR. A‐E display the mean vGRF waveforms; F‐J display the mean differences between the ACLR and uninvolved limbs; K‐O display the mean differences between the ACLR limb and controls; P‐T display the mean differences between uninvolved limb and controls. Columns 1 to 5 represent the preop, 2, 4, 6, and 12 months timepoint, respectively. Statistically significant differences in vGRF between groups are highlighted in purple and exist when 95% confidence intervals of mean differences do not include zero. ACLR, anterior cruciate ligament reconstruction; BW, body weight; vGRF, vertical ground reaction force; preop, preoperative.

The ACLR limb demonstrated lesser vGRF during early stance at preop (7–24% of stance phase), 2 (5–26%), 4 (9–25%), and 6 months (17–23%) as well as during late stance at 2 (75–90%) and 4 months (78–89%) compared with the uninvolved limb. No differences between limbs were present at 12 months post‐ACLR (Figures 2A‐J; Table 3).

**Table 3:** Output of the waveform analyses for vGRF at preop, 2, 4, 6, and 12 months post‐ACLR between the (a) ACLR and uninvolved limb, (b) ACLR limb and controls, and (c) uninvolved limb and controls, including the areas of gait stance with significant differences, the maximum differences that occurred within those areas as well as the mean Cohen's *d* effect sizes for significant areas. Small effect size: Cohen's *d* ≤ 0.2; medium effect size: 0.2 *<* Cohen's *d* ≤ 0.5; large effect size: Cohen's *d* ≥ 0.8.^28^

Comparison	Timepoint	Areas of differences (%)	Maximum difference (BW)	Cohen's *d* effect size
Uninvolved‐ACLR	preop	7–24	0.12	1.16
	2 months	5–26	0.16	1.53
		75–90	0.09	1.24
	4 months	9–25	0.09	0.82
		78–89	0.06	1.01
	6 months	17–23	0.06	0.60
	12 months	–	–	–
Control‐ACLR	preop	6–28	0.20	1.38
		39–63	−0.06	−1.61
		75–88	0.13	1.25
	2 months	6–30	0.19	1.92
		39–63	−0.12	−1.71
		75–90	0.09	1.38
	4 months	8–30	0.12	1.26
		43–62	−0.08	−1.22
		77–88	0.07	1.04
	6 months	10–29	0.10	1.07
		44–61	−0.08	−1.14
	12 months	11–28	0.08	0.85
		47–59	−0.06	−0.90
Control‐uninvolved	preop	43–63	−0.10	−1.25
	2 months	23–29	0.06	0.78
		44–59	−0.08	−1.12
	4 months	48–55	−0.06	−0.78
	6 months	49–55	−0.06	−0.76
	12 months	–	–	–

Abbreviations: ACLR, anterior cruciate ligament reconstruction; BW, body weight; preop, preoperative.

##### ACLR limb versus controls

The ACLR limb demonstrated lesser vGRF during early stance and greater vGRF during midstance at all timepoints compared with controls (preop: 6–28%, 39–63%; 2 months: 6–30%, 39–63%; 4 months: 8–30%, 43–62%; 6 months: 10–29%, 44–61%; 12 months: 11–28%, 47–59%). Additionally, the ACLR limb exhibited lesser vGRF at preop (75–88%), 2 (75–90%), and 4 months (77–88%) post‐ACLR during late stance (Figures 2A‐E, 2K‐O; Table 3).

##### Uninvolved limb versus controls

The uninvolved limb demonstrated greater vGRF during mid‐stance at preop (43–63%), 2 (44–59%), 4 (48–55%), and 6 months (49–55%) post‐ACLR and lesser vGRF during early stance at 2 months (23–29%) post‐ACLR compared with the controls. There were no differences between groups at 12 months post‐ACLR (Figures 2A‐E, 2P‐T; Table 3).

#### 3.2.2 Knee flexion angle

##### ACLR limb versus uninvolved limb

**Figure 3:**
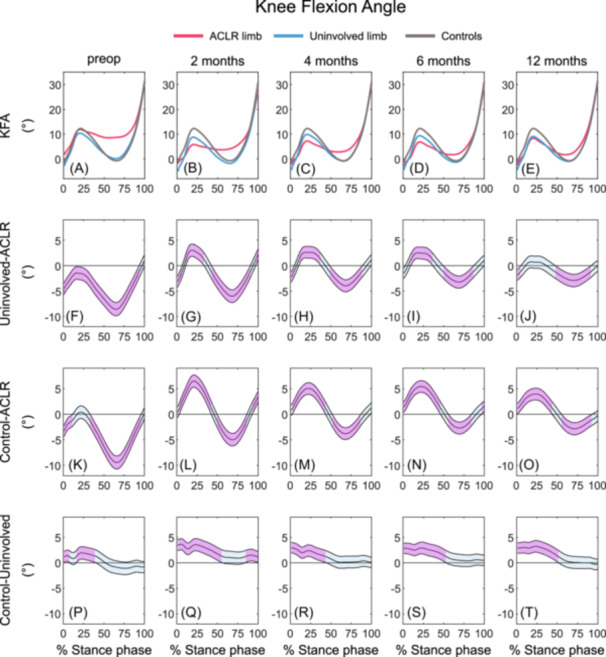
Mean KFA waveforms and mean KFA differences with 95% confidence intervals (light blue areas) throughout gait stance in the ACLR limb, uninvolved limb, and uninjured control limb at preop, 2, 4, 6, and 12 months post‐ACLR. A‐E display the mean KFA waveforms; F‐J display the mean differences between the ACLR and uninvolved limbs; K‐O display the mean differences between the ACLR limb and controls; P‐T display the mean differences between uninvolved limb and controls. Columns 1 to 5 represent the preop, 2, 4, 6, and 12 months timepoint, respectively. Statistically significant differences in KFA between groups are highlighted in purple and exist when 95% confidence intervals of mean differences do not include zero. ACLR, anterior cruciate ligament reconstruction; KFA, knee flexion angle; preop, preoperative.

The ACLR limb displayed greater KFA during the majority of stance at preop (1–93%) compared with the uninvolved limb. KFA during early stance was smaller in the ACLR limb at 2 (12–33%), 4 (11–37%), and 6 months (10–36%) compared with the uninvolved limb, whereas greater KFA during early stance at 2 (1–6%), 4 (1–4%), and 12 months (1–5%) and greater KFA during mid‐ to late stance was found in the ACLR limb compared with the uninvolved limb at 2 (43–90%), 4 (49–88%), 6 (50–86%), and 12 months (48–91%) post‐ACLR (Figures 3A‐J; Table 4).

**Table 4:** Output of the waveform analyses for KFA at preop, 2, 4, 6, and 12 months post‐ACLR between the (a) ACLR and uninvolved limb, (b) ACLR limb and controls, and (c) uninvolved limb and controls, including the areas of gait stance with significant differences, the maximum differences that occurred within those areas as well as the mean Cohen's *d* effect sizes for significant areas. Small effect size: Cohen's *d* ≤ 0.2; medium effect size: 0.2 < Cohen's *d* ≤ 0.5; large effect size: Cohen's *d* ≥ 0.8.^28^

Comparison	Timepoint	Areas of differences (%)	Maximum difference (°)	Cohen's *d* effect Size
Uninvolved‐ACLR	preop	1–93	−8.58	−1.71
	2 months	1–6	−3.17	−0.85
		12–33	3.04	0.52
		43–90	−6.00	−1.35
		97–100	2.54	0.50
	4 months	1–4	−2.26	−0.59
		11–37	2.26	0.45
		49–88	−3.95	−0.94
	6 months	10–36	2.53	0.47
		50–86	−3.22	−0.83
	12 months	1–5	−1.85	−0.51
		48–91	−2.92	−0.64
Control‐ACLR	preop	1–11	−3.30	−0.75
		34–95	−9.43	−1.79
	2 months	3–42	6.49	1.12
		50–86	−4.99	−1.05
		93–100	3.72	0.72
	4 months	3–43	5.04	0.88
		52–86	−3.80	−0.83
	6 months	1–46	5.43	0.96
		59–84	−2.70	−0.62
		97–100	1.71	0.37
	12 months	1–43	3.95	0.71
		56–92	−2.84	−0.61
Control‐uninvolved	preop	1–8	1.43	0.32
		16–40	1.97	0.34
	2 months	1–56	3.68	2.58
		84–100	1.54	0.35
	4 months	1–44	3.03	0.71
	6 months	1–53	2.93	0.54
	12 months	1–52	3.26	0.57

Abbreviations: ACLR, anterior cruciate ligament reconstruction; preop, preoperative.

##### ACLR limb versus controls

At preop, the ACLR limb demonstrated greater KFA during the majority of stance compared with controls (1‐11%, 34‐95%), whereas smaller KFA during the first 50% of stance and greater KFA during mid‐ to late stance was observed at 2 (3‐42%, 50‐86%), 4 (3‐43%, 52‐86%), 6 (1‐46%, 59‐84%), and 12 months (1‐43%, 56‐92%) post‐ACLR in the ACLR limb compared with controls. In addition, the ACLR limb demonstrated greater KFA during terminal stance at 2 (93‐100%) and 6 months (97‐100%) post‐ACLR (Figures 3A‐E, 3K‐O; Table 4).

##### Uninvolved limb versus controls

KFA in the uninvolved limb was lesser within the first 50% of stance compared with controls throughout all timepoints (preop: 1‐8%, 16‐40%; 2 months: 1‐56%; 4 months: 1‐44%; 6 months: 1‐53%; 12 months: 1‐52%). At 2 months (84‐100%), the uninvolved limb also demonstrated smaller KFA during terminal stance (Figures 3A‐E, 3P‐T; Table 4).

#### 3.2.3 Knee extension moment

##### ACLR limb versus uninvolved limb

**Figure 4:**
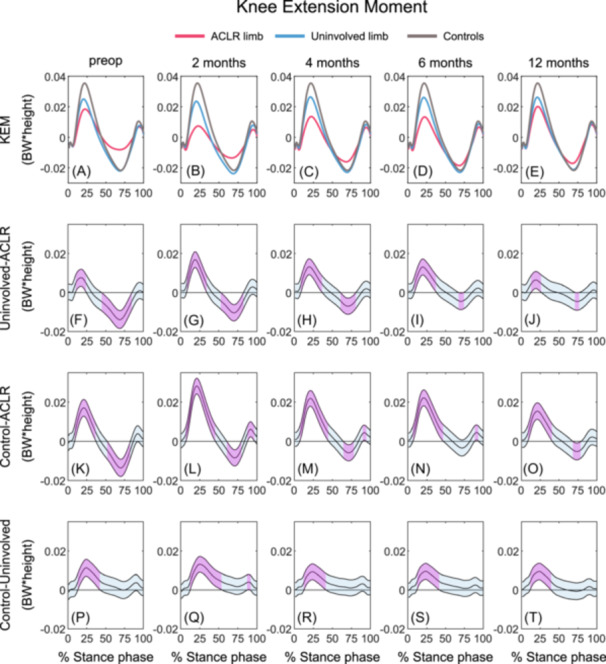
Mean KEM waveforms and mean KEM differences with 95% confidence intervals (light blue areas) throughout gait stance in the ACLR limb, uninvolved limb, and uninjured control limb at preop, 2, 4, 6, and 12 months post‐ACLR. A‐E display the mean KEM waveforms; F‐J display the mean differences between the ACLR and uninvolved limbs; K‐O display the mean differences between the ACLR limb and controls; P‐T display the mean differences between uninvolved limb and controls. Columns 1 to 5 represent the preop, 2, 4, 6, and 12 months timepoint, respectively. Statistically significant differences in KEM between groups are highlighted in purple and exist when 95% confidence intervals of mean differences do not include zero. ACLR, anterior cruciate ligament reconstruction; BW, body weight; KEM, knee extension moment; preop, preoperative.

The ACLR limb demonstrated lesser KEM during early stance as well as a lesser knee flexion moment (KFM) during mid‐to late stance at all timepoints compared with the uninvolved limb (preop: 10‐24%, 45‐84%; 2 months: 8‐34%, 53‐82%; 4 months: 9‐36%, 61‐80%; 6 months: 9‐37%, 68‐73%; 12 months: 13‐26%, 72‐76%; Figures 4A‐J; Table 5).

**Table 5:** Output of the waveform analyses for KEM at preop, 2, 4, 6, and 12 months post‐ACLR between the (a) ACLR and uninvolved limb, (b) ACLR limb and controls, and (c) uninvolved limb and controls, including the areas of gait stance with significant differences, the maximum differences that occurred within those areas as well as the mean Cohen's *d* effect sizes for significant areas. Small effect size: Cohen's *d* ≤ 0.2; medium effect size: 0.2 < Cohen's *d* ≤ 0.5; large effect size: Cohen's *d* ≥ 0.8.^28^

Comparison	Timepoint	Areas of differences (%)	Maximum difference (BW*height)	Cohen's *d* effect size
Uninvolved‐ACLR	preop	10–24	0.01	0.63
		45–84	−0.01	−1.27
	2 months	8–34	0.02	1.43
		53–82	−0.01	−1.15
	4 months	9–36	0.01	0.98
		61–80	−0.01	−0.90
	6 months	9–37	0.01	1.00
		68–73	−0.01	−0.54
	12 months	13–26	0.01	0.48
		72–76	−0.01	−0.50
Control‐ACLR	preop	9–37	0.02	1.42
		52–82	−0.01	−1.24
	2 months	7–45	0.03	2.38
		61–79	−0.01	−0.96
		88–97	0.01	1.27
	4 months	8–45	0.02	1.77
		65–78	−0.01	−0.70
		90–94	0.01	0.91
	6 months	7–47	0.02	1.75
		89–94	0.01	0.83
	12 months	9–40	0.02	1.18
		68–79	−0.01	−0.60
Control‐uninvolved	preop	14–42	0.01	0.80
	2 months	13–54	0.01	0.97
		88–91	0.01	0.64
	4 months	13–41	0.01	0.64
	6 months	12–41	0.01	0.67
	12 months	12–39	0.01	0.67

Abbreviations: ACLR, anterior cruciate ligament reconstruction; BW, body weight; preop, preoperative.

##### ACLR limb versus controls

Lesser KEM was observed for the ACLR limb during the first 50% of stance at all timepoints between preop and 12 months (preop: 9–37%; 2 months: 7–45%; 4 months: 8–45%; 6 months: 7–47%); 12 months (9–40%) as well as during terminal stance at 2 (88–97%), 4 (90–94%), and 6 months (89–94%) compared with controls. Lesser KEM was observed during mid‐ to late stance in the ACLR limb at preop (52–82%), 2 (61–79%), 4 (65–78%), and 12 months (68–79%) post‐ACLR (Figures 4A‐E, 4K‐O; Table 5).

##### Uninvolved limb versus controls

For all timepoints, the uninvolved limb demonstrated lesser KEM during early stance compared with controls (preop: 14–42%; 2 months: 13–54%; 4 months: 13–41%; 6 months: 12–41%; 12 months: 12–39%; Figures 4A‐E, 4P‐T; Table 5). In addition, lesser KEM was observed during late stance at 2 months (88–91%) post‐ACLR in the uninvolved limb compared with controls.

#### 3.2.4 Knee adduction moment

##### ACLR limb versus uninvolved limb

**Figure 5:**
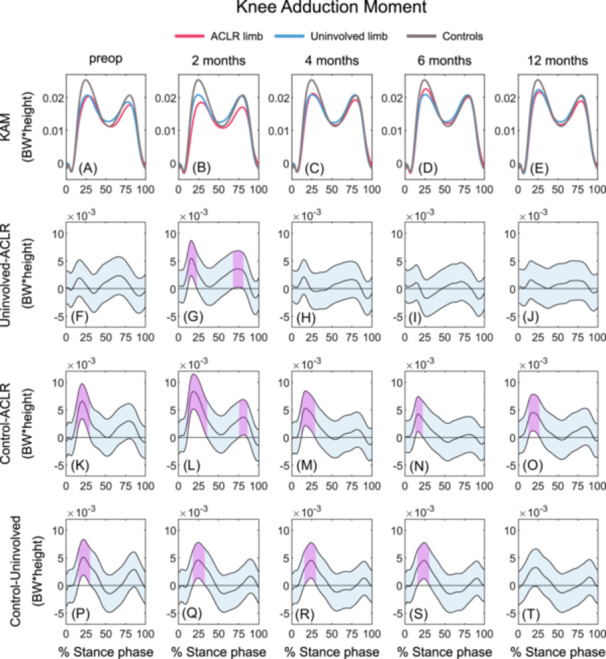
Mean KAM waveforms and mean KAM differences with 95% confidence intervals (light blue areas) throughout gait stance in the ACLR limb, uninvolved limb, and uninjured control limb at preop, 2, 4, 6, and 12 months post‐ACLR. A‐E display the mean KAM waveforms; F‐J display the mean differences between the ACLR and uninvolved limbs; K‐O display the mean differences between the ACLR limb and controls; P‐T display the mean differences between uninvolved limb and controls. Columns 1 to 5 represent the preop, 2, 4, 6, and 12 months timepoint, respectively. Statistically significant differences in KAM between groups are highlighted in purple and exist when 95% confidence intervals of mean differences do not include zero. ACLR, anterior cruciate ligament reconstruction; BW, body weight; KAM, knee adduction moment; preop, preoperative.

Lesser KAM was observed in the ACLR limb during early and late stance at 2 months (12–23%, 69–80%) compared with the uninvolved limb. No differences between limbs were observed at preop, 4, 6, and 12 months post‐ACLR (Figures 5A‐J; Table 6).

**Table 6:** Output of the waveform analyses for KAM at preop, 2, 4, 6, and 12 months post‐ACLR between the (a) ACLR and uninvolved limb, (b) ACLR limb and controls, and (c) uninvolved limb and controls, including the areas of gait stance with significant differences, the maximum differences that occurred within those areas as well as the mean Cohen's *d* effect sizes for significant areas. Small effect size: Cohen's *d* ≤ 0.2; medium effect size: 0.2 < Cohen's *d* ≤ 0.5; large effect size: Cohen's *d* ≥ 0.8.^28^

Comparison	Timepoint	Areas of differences (%)	Maximum difference (BW*height)	Cohen's *d* effect size
Uninvolved‐ACLR	preop	–	–	–
	2 months	12‐23	0.01	0.79
		69‐80	0.01	0.45
	4 months	–	–	–
	6 months	–	–	–
	12 months	–	–	–
Control‐ACLR	preop	14‐30	0.01	0.79
	2 months	12‐35	0.01	1.12
		76‐85	0.01	0.46
	4 months	12‐29	0.01	0.73
	6 months	13‐22	0.01	0.60
	12 months	13‐26	0.01	0.61
Control‐uninvolved	preop	16‐31	0.01	0.67
	2 months	18‐33	0.01	0.58
	4 months	16‐30	0.01	0.60
	6 months	16‐30	0.01	0.56
	12 months	–	–	–

Abbreviations: ACLR, anterior cruciate ligament reconstruction; BW, body weight; preop, preoperative.

##### ACLR limb versus controls

The ACLR limb demonstrated lesser KAM during early stance at all time points (preop: 14–30%; 2 months: 12–35%; 4 months: 12–29%; 6 months: 13–22%, 12 months: 13–26%) compared with controls (Figures 5A‐E, 5K‐O; Table 6). In addition, lesser KAM was observed during late stance at 2 months post‐ACLR in the ACLR‐limb compared with controls.

##### Uninvolved limb versus controls

The uninvolved limb had lesser KAM during early stance at timepoints between preop and 6 months (preop: 16–31%; 2 months: 18–33%; 4 months: 16–30%, 6 months: 16–30%) compared with controls, but no differences were observed at 12 months post‐ACLR (Figures 5A‐E, 5P‐T; Table 6).

#### 3.2.5 Differences in gait biomechanical outcomes with gait speed adjustment

The detailed results of the of the waveform comparisons while adjusting for gait speed can be found in Supplement 2 (Figures S2.1–4; Tables S2.1–4). Overall, the analysis with gait speed adjustment demonstrated similar outcomes as those of the non‐adjusted analysis. After adjusting for gait speed, no differences were found for vGRF at 6 and 12 months post‐ACLR and no midstance differences at all timepoints between the ACLR and uninvolved limbs. In addition, greater vGRF was found in the uninvolved limb during early stance at preop and 2 months post‐ACLR compared with controls. The adjustment for gait speed led to marginal changes in the interpretations for between limb and group effects for KFA. Deviations from the analyses without gait speed adjustment included no differences during early stance at preop and late stance at 2 months post‐ACLR in the uninvolved limb compared with controls, and greater KFA during terminal stance at the preop timepoint in the uninvolved limb compared with controls. The results for the KEM are also consistent with the analyses without gait speed adjustment, except no differences were present in late stance at 2, 4, and 6 months between the ACLR limb and controls as well as between the uninvolved limb and controls at preop and 4 months post‐ACLR. Adjusting for gait speed limited the statistically significant differences to lesser KAM during early and late stance at 2 months post‐ACLR between the ACLR limb and the uninvolved limb and lesser KAM during early stance at 2 months post‐ACLR in the ACLR limb compared with controls.

## 4 DISCUSSION

Consistent with our hypotheses, individuals with primary unilateral ACLR demonstrated different bilateral gait biomechanics (i.e., vGRF, KFA, KEM, and KAM) at habitual walking speeds compared to age‐, sex‐, and BMI‐matched controls between preop and 12 months post‐ACLR. While the ACLR and uninvolved limbs became more symmetrical over time in all outcome variables, differences between the ACLR limb and matched controls are greater than the differences between the uninjured limb and matched controls (Tables 2, 3, 4, 5). Our data indicate that the biomechanics of the ACLR limb are, as expected, more impacted than the uninjured limb by injury and surgery within the first 12 months post‐ACLR. These results indicate that bilateral aberrant gait biomechanics are apparent before ACLR and persist beyond the time at which most individuals have completed formal rehabilitation and, in many cases, have already returned to unrestricted physical activity.^14^, ^37^ Given the persistent bilateral changes in gait biomechanics within the first 12 months post‐ACLR, there is a critical need to implement early precision gait retraining following ACLR in the involved and uninvolved limbs to restore gait biomechanics, which could help maintaining long‐term knee joint health following ACL injury.

Overall, biomechanical waveform profiles collected at habitual walking speeds for vGRF, KFA, KEM, KAM at all time points between preop and 12 months post‐ACLR can be characterized as less dynamic in the ACLR group compared with matched controls. A less dynamic waveform can be described as exhibiting smaller peaks and an overall flatter appearance in time‐normalized graphical waveform representations. Less dynamic vGRF waveforms have been reported in individuals with ACLR as well as those with KOA,^19^, ^38^, ^39^ and are linked to outcomes related to KOA development, including worse cartilage composition, deleterious joint tissue metabolism, and worse patient reported outcomes.^2^, ^6^, ^8^, ^12^ It is hypothesized that less dynamic vGRF waveforms may result in more sustained compressive loading to the knee joint, which in combination with the viscoelastic nature of joint tissues like articular cartilage,^40^ may result in greater tissue strain that could perpetuate joint tissue breakdown. Furthermore, less dynamic KAM as seen within in the ACLR group has also been linked to KOA development post‐ACLR.^10^, ^41^ Our study also found that the ACLR limb demonstrated less dynamic KFA and KEM throughout stance with lesser peak KFA and KEM in early stance and greater knee flexion in midstance post‐ACLR compared to controls. A stiffened‐knee gait during stance, caused by lesser overall knee range of motion,^19^ may alter tibiofemoral and patellofemoral contact force profiles and localize loading to specific areas of the joint.^42^, ^43^ It can be hypothesized that the combination of more sustained and localized joint loading may contribute to deleterious changes in joint tissue health that result in the development of KOA.

We found that primary unilateral ACL injury and ACLR led to bilateral gait alterations when collected at habitual walking speeds. Over time, asymmetries between the ACLR and uninvolved limbs mostly resolve with only marginal differences in KFA and KEM being present by the 12 months timepoint. Increased limb symmetry in gait biomechanics over time has been described in previous literature.^16^, ^17^, ^19^ However, Davis‐Wilson et al.^19^ reported that the uninvolved side seem to develop more aberrant gait biomechanics from 6 to 12 months, by adopting biomechanical patterns more similar to the uninjured limb instead of gait outcomes similar to uninjured controls. However, in the current study, gait biomechanics in the uninvolved limb improved (in vGRF, KAM) or were stable (in KFA, KEM) over time compared to controls from preop to 12 months post‐ACLR and differences between the ACLR and uninvolved limb decreased from 6 to 12 months. Despite improvements in gait over the first 12 months post‐ACLR and improvements in limb symmetry, the uninvolved limb still does not exhibit normalized gait biomechanics, as defined by our control group, by the 12‐month timepoint. Therefore, gait rehabilitation following unilateral ACLR should include bilateral intervention. The need for bilateral rehabilitation is highlighted by research demonstrating that the uninvolved limb also undergoes deleterious cartilage compositional changes that are associated with early KOA development.^18^, ^22^ Yet, it is not fully understood what causes the biomechanical and biological alterations in the uninvolved limb. A slower gait speed, as seen in our ACLR cohort as well as altered neuromuscular function following ACLR could contribute to bilateral changes in gait biomechanics,^44^ which could negatively affect cartilage composition. Given the biomechanical and biological changes in the uninvolved limb following ACL injury, the uninvolved limb should not be used as the only reference‐control limb in future studies investigating knee‐specific kinematics and kinetics during the stance phase of gait since analytical approaches may lead to misinterpretation of results.

Differences in biomechanical key variables are commonly reported for peak outcomes within the first 50% of stance post‐ACLR. Our results demonstrate that differences between limbs and controls may also exist during midstance in vGRF as well as early to late stance in KFA. While aberrant peak values within the first 50% of stance are associated with outcomes linked to KOA development post‐ACLR,^2^, ^4^, ^8^, ^9^, ^10^, ^45^, ^46^ initial research has demonstrated that limb loading at or around midstance might be more predictive of knee‐related cartilage changes associated with KOA following ACLR than peak vGRF in individuals 12 months post‐ACLR.^6^ Thus, only evaluating peak outcomes within the first half of stance may lead to poor interpretations of results. Therefore, future research should investigate if portions of stance other than the first peak (e.g., 2nd peak KAM and vGRF, peak KFM, KFA at heel contact, midstance and terminal stance) are associated with later KOA development. Identifying the portions of stance with the strongest link to KOA development post‐ACLR for key biomechanical variables would help the development of new KOA prevention gait therapies, such as real‐time gait biofeedback,^26^, ^27^ which could target the most influential portions of stance to prevent joint breakdown.

Gait speed is known to influence gait biomechanics, with faster speeds associated with greater peak vGRF, greater peak knee moments and greater knee flexion angles.^44^, ^47^, ^48^, ^49^ In our study, the ACLR group walked at slower habitual speeds at all 5 timepoints compared to uninjured controls (mean gait speed ACLR group: 1.16–1.27 m/s, controls: 1.36 m/s, Table 1). We chose to report our results at each participant's habitual walking speed as measuring biomechanics at habitual gait speed may be more representative of the movements that are occurring in the real‐world and assist in preserving a degree of externally validity when determining differences in gait biomechanics between limbs and as compared to uninjured controls at each timepoint. There were marginal differences in the outputs of our analyses with and without adjusting for gait speed, but both approaches demonstrate that differences in gait biomechanics between the ACLR limb, uninvolved limb, and controls persist up until 12 months post‐ACLR. In particular, when controlling for gait speed, we did not find differences in vGRF and KAM between the ACLR limb and the uninvolved or control limbs at 12 months post‐ACLR. However, differences between the ACLR limb, uninvolved limb, and controls existed for KFA and KEM even after controlling for gait speed. Overall, our analyses with gait speed adjustment suggest that slower walking speed may have been a mechanism contributing to some of the differences between limbs that were found in our analyses without adjusting for gait speed. Further, while statistically correcting for walking speed resulted in slightly different results between comparisons, recent work suggests that increasing gait speed in slow walkers with an ACLR does not result in normalized gait biomechanics post‐ACLR.^30^ However normalizing gait speed in addition to cueing changes in other gait biomechanics may be an important part of a comprehensive rehabilitation strategy to restore overall gait post‐ACLR, since slower speeds have been associated with worse cartilage composition and cartilage breakdown related to KOA development.^35^, ^36^

While this is the first comprehensive waveform analysis of gait biomechanics from preop to 12 months post‐ACLR including the ACLR limb, uninvolved limb, and uninjured controls, there are limitations of our study that should be considered for future research. We only included individuals with unilateral ACL injury of which most (93%) received a bone‐patellar‐bone tendon autograft. Therefore, our results are not generalizable to individuals who received different graft types, had multiple ACL injuries/surgeries, or are ACL‐deficient. We also did not control the rehabilitation following injury/surgery, however, including individuals who received general postoperative care makes the results of our study more generalizable to the current standard of care. We assessed gait biomechanics up until 12 months post‐ACLR and can therefore not determine whether aberrant bilateral gait biomechanics persist beyond 12 months post‐ACLR. Since age can impact gait biomechanical outcomes following ACLR,^32^ our results may not be generalizable to individuals with ACLR that are younger than 16 or older than 35 years old. Furthermore, it is unknown whether there were natural differences in gait biomechanics and gait speed between the ACLR group and controls before ACL injury.

Overall, gait biomechanics, collected at habitual walking speeds, are changed bilaterally between preoperative and 12 months post‐ACLR in vGRF, KFA, KEM, and KAM compared with controls. Though the ACLR and uninvolved limbs become more symmetrical over time, the ACLR limb persistently demonstrates greater differences in gait biomechanics compared to controls than the uninvolved limb. Generally, gait biomechanics following ACLR can be characterized as less dynamic than matched controls, which has been linked to KOA development and progression. Our results indicate that early bilateral precision interventions are needed to normalize gait, which could improve long‐term knee joint health outcomes following ACLR.

## AUTHOR CONTRIBUTION

All authors contributed to a combination of research design, data acquisition, analysis, and interpretation, as well as drafting and critical revision of the manuscript. All authors have read and approved the final submitted manuscript.

## Supporting information

Supporting information.

Supporting information.
