# Supplementary material for: Bilateral waveform analysis of gait biomechanics presurgery to 12 months following ACL reconstruction compared to controls
Source: J Orthop Res. 2024 Dec 4;43(2):322–36. doi: 10.1002/jor.26001 (PMC11701409; doi:10.1002/jor.26001)
Supplement: Supplementary file 1 — Supporting information. [file JOR-43-322-s001.docx]

Table S1.1: Contribution of each study participant to the biomechanics analyses at each timepoint. x indicates the biomechanical data was available for analyses; areas highlighted in light red indicate that the data was not available for analyses.

| Subject |  | Preop (n=50) | | | | | 2 months (n=54) | | | | | | 4 months (n=55) | | | | | | 6 months (n=53) | | | | | | 12 months (n=50) | | | | | |
| --- | --- | --- | --- | --- | --- | --- | --- | --- | --- | --- | --- | --- | --- | --- | --- | --- | --- | --- | --- | --- | --- | --- | --- | --- | --- | --- | --- | --- | --- | --- |
|  |  | vGRF | KFA | KEM | KAM |  | | vGRF | KFA | KEM | KAM |  | | vGRF | KFA | KEM | KAM |  | | vGRF | KFA | KEM | KAM |  | | vGRF | KFA | KEM | KAM |  |
| S1 |  | x | x | x | x |  | | x | x | x | x |  | | x | x | x | x |  | | x | x | x | x |  | |  |  |  |  |  |
| S2 |  |  |  |  |  |  | | x | x | x | x |  | |  |  |  |  |  | |  |  |  |  |  | | x | x | x | x |  |
| S3 |  | x | x | x | x |  | | x | x | x | x |  | | x | x | x | x |  | |  |  |  |  |  | |  |  |  |  |  |
| S4 |  | x | x | x | x |  | | x | x | x | x |  | | x | x | x | x |  | | x | x | x | x |  | | x | x | x | x |  |
| S5 |  |  |  |  |  |  | | x | x | x | x |  | | x | x | x | x |  | |  |  |  |  |  | | x | x | x | x |  |
| S6 |  |  |  |  |  |  | |  |  |  |  |  | |  |  |  |  |  | |  |  |  |  |  | |  |  |  |  |  |
| S7 |  | x | x | x | x |  | |  |  |  |  |  | | x | x | x | x |  | | x | x | x | x |  | | x | x | x | x |  |
| S8 |  | x | x | x | x |  | | x | x | x | x |  | | x | x | x | x |  | | x | x | x | x |  | | x | x | x | x |  |
| S9 |  | x | x | x | x |  | | x | x | x | x |  | | x | x | x | x |  | | x | x | x | x |  | | x | x | x | x |  |
| S10 |  |  |  |  |  |  | | x | x | x | x |  | | x | x | x | x |  | | x | x | x | x |  | | x | x | x | x |  |
| S11 |  | x | x | x | x |  | | x | x | x | x |  | | x | x | x | x |  | | x | x | x | x |  | | x | x | x | x |  |
| S12 |  | x | x | x | x |  | | x | x | x | x |  | | x | x | x | x |  | | x | x | x | x |  | | x | x | x | x |  |
| S13 |  | x | x | x | x |  | | x | x | x | x |  | |  |  |  |  |  | | x | x | x | x |  | | x | x | x | x |  |
| S14 |  | x | x | x | x |  | | x | x | x | x |  | | x | x | x | x |  | | x | x | x | x |  | | x | x | x | x |  |
| S15 |  | x | x | x | x |  | | x | x | x | x |  | | x | x | x | x |  | | x | x | x | x |  | | x | x | x | x |  |
| S16 |  | x | x | x | x |  | | x | x | x | x |  | | x | x | x | x |  | | x | x | x | x |  | | x | x | x | x |  |
| S17 |  |  |  |  |  |  | | x | x | x | x |  | | x | x | x | x |  | | x | x | x | x |  | | x | x | x | x |  |
| S18 |  |  |  |  |  |  | | x | x | x | x |  | | x | x | x | x |  | | x | x | x | x |  | | x | x | x | x |  |
| S19 |  |  |  |  |  |  | | x | x | x | x |  | | x | x | x | x |  | | x | x | x | x |  | |  |  |  |  |  |
| S20 |  | x | x | x | x |  | | x | x | x | x |  | | x | x | x | x |  | | x | x | x | x |  | | x | x | x | x |  |
| S21 |  |  |  |  |  |  | | x | x | x | x |  | | x | x | x | x |  | | x | x | x | x |  | | x | x | x | x |  |
| S22 |  | x | x | x | x |  | | x | x | x | x |  | | x | x | x | x |  | | x | x | x | x |  | | x | x | x | x |  |
| S23 |  | x | x | x | x |  | | x | x | x | x |  | | x | x | x | x |  | | x | x | x | x |  | | x | x | x | x |  |
| S24 |  |  |  |  |  |  | |  |  |  |  |  | |  |  |  |  |  | |  |  |  |  |  | |  |  |  |  |  |
| S25 |  | x | x | x | x |  | | x | x | x | x |  | | x | x | x | x |  | | x | x | x | x |  | | x | x | x | x |  |
| S26 |  | x | x | x | x |  | | x | x | x | x |  | | x | x | x | x |  | | x | x | x | x |  | | x | x | x | x |  |
| S27 |  | x | x | x | x |  | | x | x | x | x |  | | x | x | x | x |  | | x | x | x | x |  | | x | x | x | x |  |
| S28 |  | x | x | x | x |  | | x | x | x | x |  | | x | x | x | x |  | | x | x | x | x |  | | x | x | x | x |  |
| S29 |  | x | x | x | x |  | | x | x | x | x |  | | x | x | x | x |  | | x | x | x | x |  | | x | x | x | x |  |
| S30 |  | x | x | x | x |  | | x | x | x | x |  | | x | x | x | x |  | | x | x | x | x |  | | x | x | x | x |  |
| S31 |  | x | x | x | x |  | | x | x | x | x |  | | x | x | x | x |  | | x | x | x | x |  | | x | x | x | x |  |
| S32 |  | x | x | x | x |  | | x | x | x | x |  | | x | x | x | x |  | | x | x | x | x |  | | x | x | x | x |  |
| S33 |  | x | x | x | x |  | | x | x | x | x |  | | x | x | x | x |  | | x | x | x | x |  | | x | x | x | x |  |
| S34 |  | x | x | x | x |  | | x | x | x | x |  | | x | x | x | x |  | | x | x | x | x |  | | x | x | x | x |  |
| S35 |  | x | x | x | x |  | | x | x | x | x |  | | x | x | x | x |  | | x | x | x | x |  | | x | x | x | x |  |
| S36 |  | x | x | x | x |  | | x | x | x | x |  | | x | x | x | x |  | | x | x | x | x |  | | x | x | x | x |  |
| S37 |  | x | x | x | x |  | | x | x | x | x |  | | x | x | x | x |  | | x | x | x | x |  | |  |  |  |  |  |
| S38 |  | x | x | x | x |  | | x | x | x | x |  | | x | x | x | x |  | | x | x | x | x |  | |  |  |  |  |  |
| S39 |  |  |  |  |  |  | |  |  |  |  |  | |  |  |  |  |  | |  |  |  |  |  | |  |  |  |  |  |
| S40 |  | x | x | x | x |  | | x | x | x | x |  | | x | x | x | x |  | | x | x | x | x |  | | x | x | x | x |  |
| S41 |  | x | x | x | x |  | | x | x | x | x |  | | x | x | x | x |  | | x | x | x | x |  | |  |  |  |  |  |
| S42 |  | x | x | x | x |  | | x | x | x | x |  | | x | x | x | x |  | |  |  |  |  |  | | x | x | x | x |  |
| S43 |  | x | x | x | x |  | | x | x | x | x |  | | x | x | x | x |  | | x | x | x | x |  | | x | x | x | x |  |
| S44 |  | x | x | x | x |  | | x | x | x | x |  | | x | x | x | x |  | | x | x | x | x |  | |  |  |  |  |  |
| S45 |  | x | x | x | x |  | | x | x | x | x |  | | x | x | x | x |  | | x | x | x | x |  | | x | x | x | x |  |
| S46 |  | x | x | x | x |  | | x | x | x | x |  | | x | x | x | x |  | | x | x | x | x |  | | x | x | x | x |  |
| S47 |  |  |  |  |  |  | |  |  |  |  |  | | x | x | x | x |  | | x | x | x | x |  | | x | x | x | x |  |
| S48 |  | x | x | x | x |  | | x | x | x | x |  | | x | x | x | x |  | | x | x | x | x |  | | x | x | x | x |  |
| S49 |  | x | x | x | x |  | | x | x | x | x |  | | x | x | x | x |  | | x | x | x | x |  | | x | x | x | x |  |
| S50 |  |  |  |  |  |  | |  |  |  |  |  | |  |  |  |  |  | |  |  |  |  |  | |  |  |  |  |  |
| S51 |  | x | x | x | x |  | | x | x | x | x |  | | x | x | x | x |  | | x | x | x | x |  | | x | x | x | x |  |
| S52 |  | x | x | x | x |  | |  |  |  |  |  | | x | x | x | x |  | | x | x | x | x |  | | x | x | x | x |  |
| S53 |  | x | x | x | x |  | | x | x | x | x |  | | x | x | x | x |  | | x | x | x | x |  | | x | x | x | x |  |
| S54 |  | x | x | x | x |  | | x | x | x | x |  | | x | x | x | x |  | | x | x | x | x |  | | x | x | x | x |  |
| S55 |  | x | x | x | x |  | | x | x | x | x |  | | x | x | x | x |  | | x | x | x | x |  | | x | x | x | x |  |
| S56 |  | x | x | x | x |  | | x | x | x | x |  | | x | x | x | x |  | | x | x | x | x |  | | x | x | x | x |  |
| S57 |  | x | x | x | x |  | | x | x | x | x |  | | x | x | x | x |  | | x | x | x | x |  | | x | x | x | x |  |
| S58 |  | x | x | x | x |  | | x | x | x | x |  | | x | x | x | x |  | | x | x | x | x |  | | x | x | x | x |  |
| S59 |  | x | x | x | x |  | | x | x | x | x |  | | x | x | x | x |  | | x | x | x | x |  | | x | x | x | x |  |
| S60 |  | x | x | x | x |  | | x | x | x | x |  | | x | x | x | x |  | | x | x | x | x |  | | x | x | x | x |  |
| S61 |  | x | x | x | x |  | |  |  |  |  |  | |  |  |  |  |  | |  |  |  |  |  | |  |  |  |  |  |
| S62 |  | x | x | x | x |  | | x | x | x | x |  | | x | x | x | x |  | | x | x | x | x |  | | x | x | x | x |  |
| Preop – preoperative, vGRF – vertical ground reaction force, KFA – knee flexion angle, KEM – knee extension moment, KAM – knee adduction moment | | | | | | | | | | | | | | | | | | | | | | | | | | | | | |  |
