# Supplementary material for: Bilateral waveform analysis of gait biomechanics presurgery to 12 months following ACL reconstruction compared to controls
Source: J Orthop Res. 2024 Dec 4;43(2):322–36. doi: 10.1002/jor.26001 (PMC11701409; doi:10.1002/jor.26001)
Supplement: Supplementary file 2 — Supporting information. [file JOR-43-322-s002.docx]

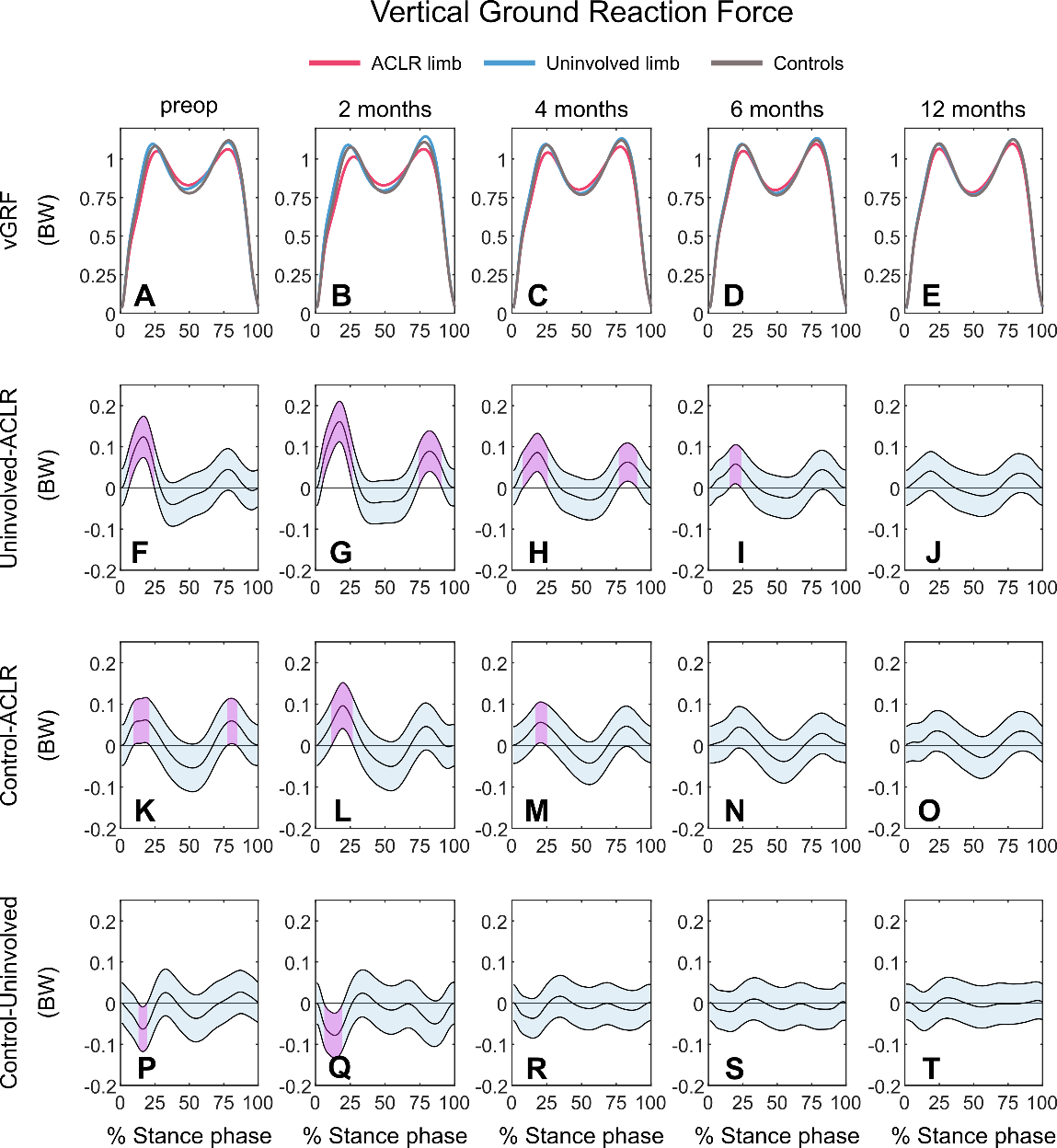


Figure S2.1: Mean vGRF waveforms and mean vGRF differences with 95% confidence intervals (light blue areas) adjusted for gait speed throughout gait stance in the ACLR limb, uninvolved limb, and uninjured control limb at preop, 2, 4, 6, and 12 months post-ACLR. A-E display the mean vGRF waveforms; F-J display the mean differences between the ACLR and uninvolved limbs; K-O display the mean differences between the ACLR limb and controls; P-T display the mean differences between uninvolved limb and controls. Columns 1 to 5 represent the preop, 2, 4, 6, and 12 months timepoint, respectively. Statistically significant differences in vGRF between groups are highlighted in purple and exist when 95% confidence intervals of mean differences do not include zero. ACLR – anterior cruciate ligament reconstruction, BW – body weight, vGRF – vertical ground reaction force, preop – preoperative


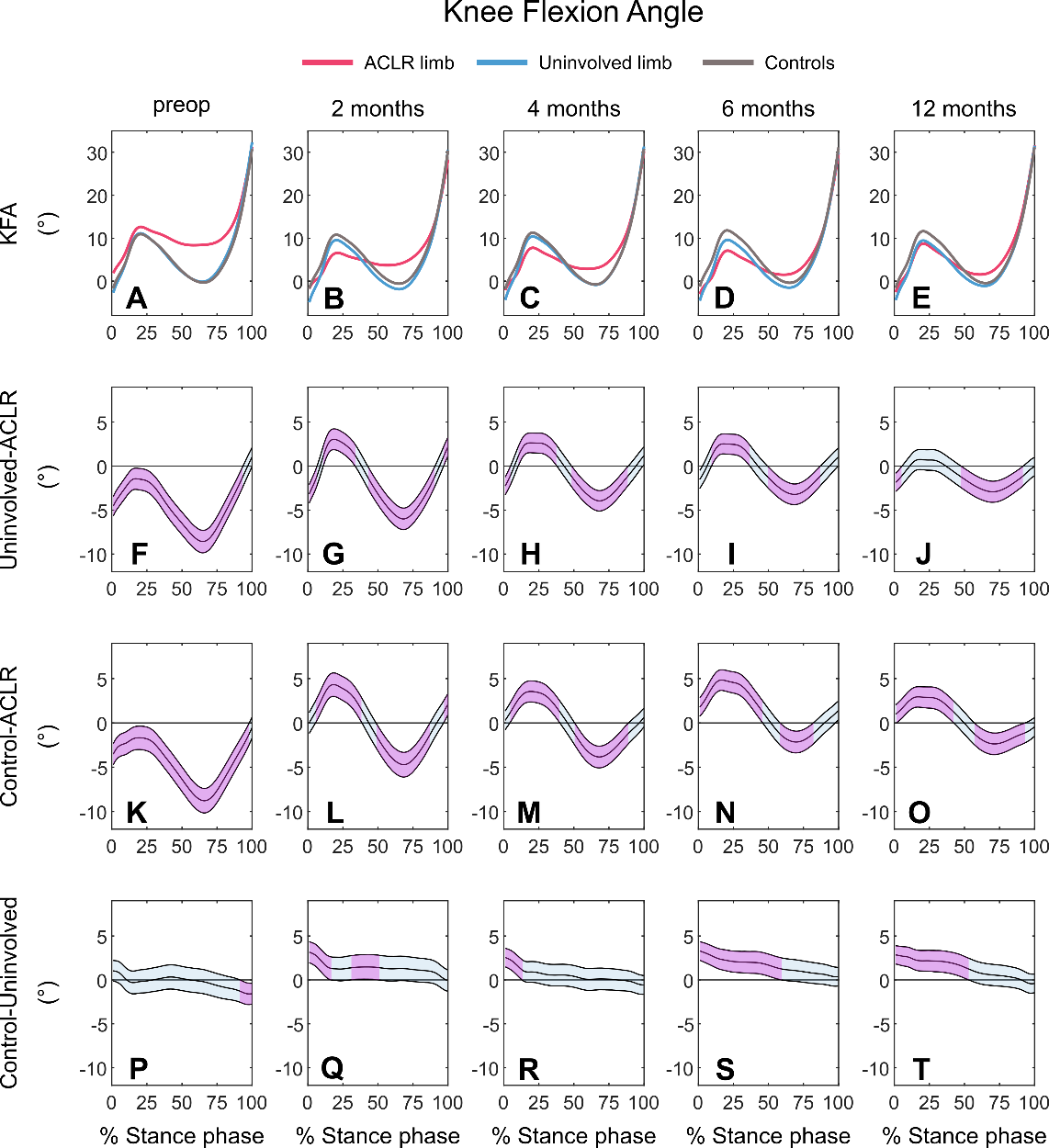


Figure S2.2: Mean KFA waveforms and mean KFA differences with 95% confidence intervals (light blue areas) adjusted for gait speed throughout gait stance in the ACLR limb, uninvolved limb, and uninjured control limb at preop, 2, 4, 6, and 12 months post-ACLR. A-E display the mean KFA waveforms; F-J display the mean differences between the ACLR and uninvolved limbs; K-O display the mean differences between the ACLR limb and controls; P-T display the mean differences between uninvolved limb and controls. Columns 1 to 5 represent the preop, 2, 4, 6, and 12 months timepoint, respectively. Statistically significant differences in KFA between groups are highlighted in purple and exist when 95% confidence intervals of mean differences do not include zero. ACLR – anterior cruciate ligament reconstruction, BW – body weight, KFA – knee flexion angle, preop – preoperative


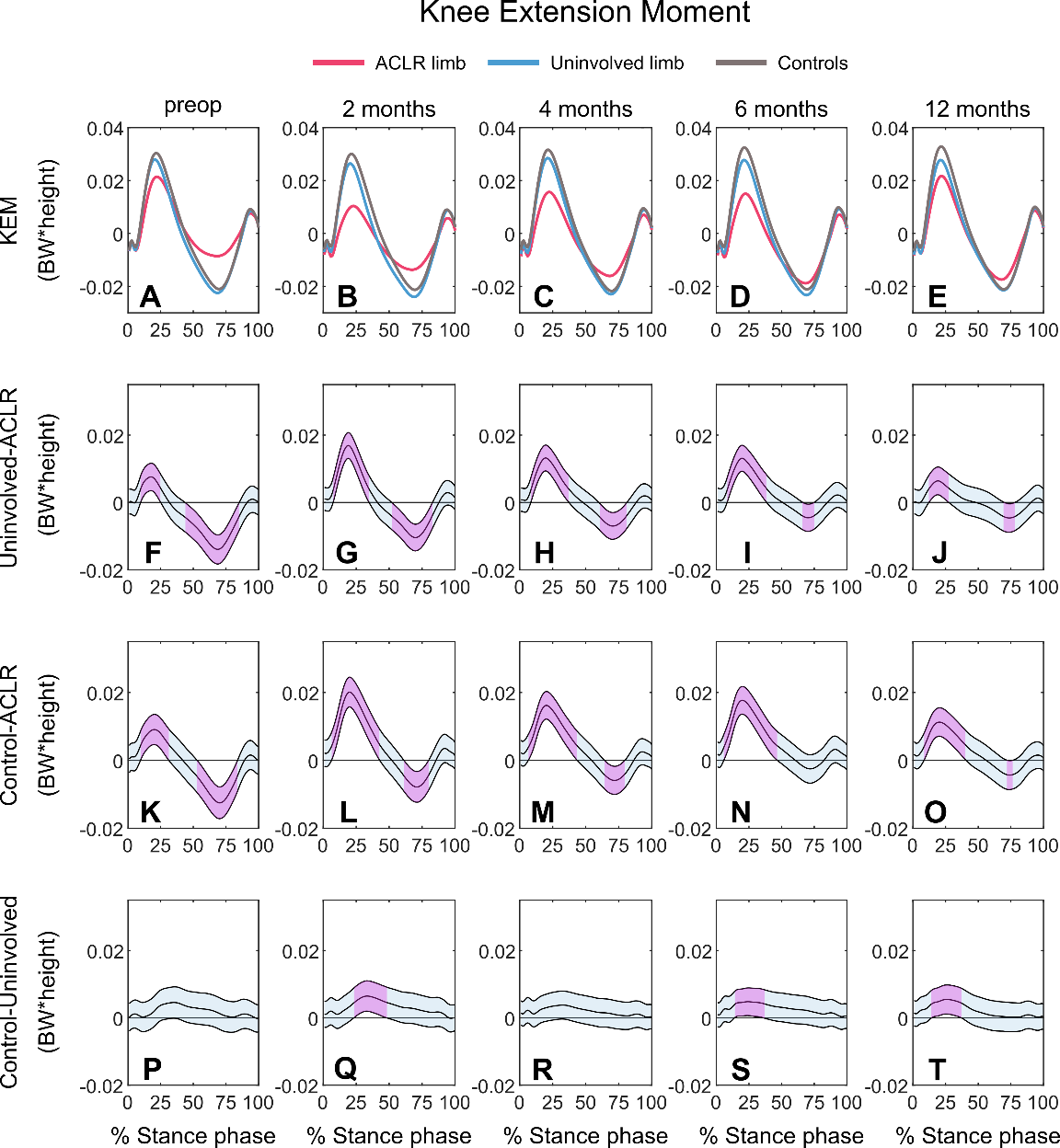


Figure S2.3: Mean KEM waveforms and mean KEM differences with 95% confidence intervals (light blue areas) adjusted for gait speed throughout gait stance in the ACLR limb, uninvolved limb, and uninjured control limb at preop, 2, 4, 6, and 12 months post-ACLR. A-E display the mean KEM waveforms; F-J display the mean differences between the ACLR and uninvolved limbs; K-O display the mean differences between the ACLR limb and controls; P-T display the mean differences between uninvolved limb and controls. Columns 1 to 5 represent the preop, 2, 4, 6, and 12 months timepoint, respectively. Statistically significant differences in KEM between groups are highlighted in purple and exist when 95% confidence intervals of mean differences do not include zero. ACLR – anterior cruciate ligament reconstruction, BW – body weight, KEM – knee extension moment, preop – preoperative


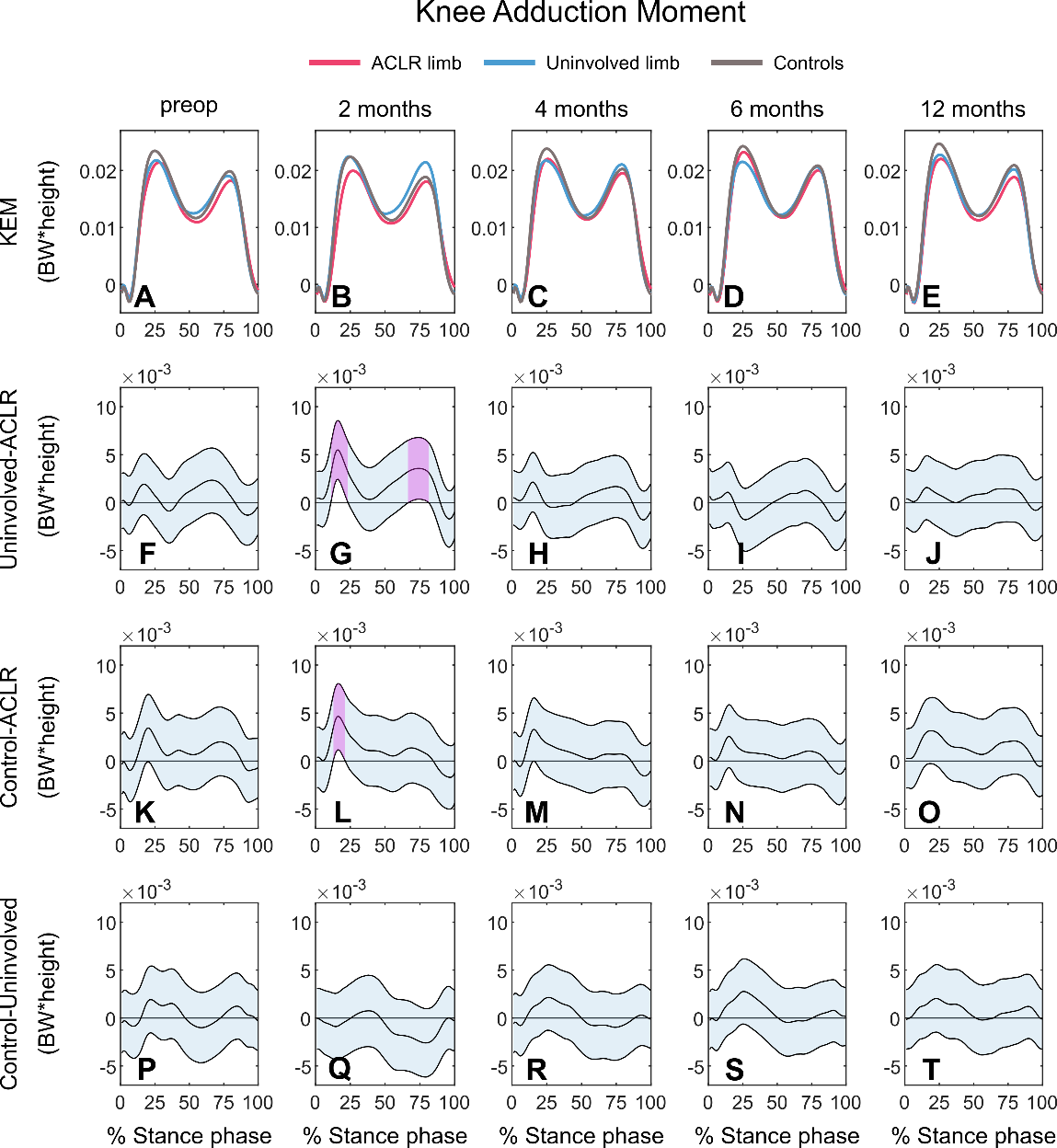


Figure S2.4: Mean KAM waveforms and mean KAM differences with 95% confidence intervals (light blue areas) adjusted for gait speed throughout gait stance in the ACLR limb, uninvolved limb, and uninjured control limb at preop, 2, 4, 6, and 12 months post-ACLR. A-E display the mean KAM waveforms; F-J display the mean differences between the ACLR and uninvolved limbs; K-O display the mean differences between the ACLR limb and controls; P-T display the mean differences between uninvolved limb and controls. Columns 1 to 5 represent the preop, 2, 4, 6, and 12 months timepoint, respectively. Statistically significant differences in KAM between groups are highlighted in purple and exist when 95% confidence intervals of mean differences do not include zero. ACLR – anterior cruciate ligament reconstruction, BW – body weight, KAM – knee adduction moment, preop – preoperative

Table S2.1: Output of the waveform analyses for vGRF after adjusting for gait speed at preop, 2, 4, 6, and 12 months post-ACLR between the a) ACLR and uninvolved limb, b) ACLR limb and controls, and c) uninvolved limb and controls, including the areas of gait stance with significant differences, the maximum differences that occurred within those areas as well as the mean Cohen’s d effect sizes for significant areas. Small effect size: Cohen’s d≤0.2; medium effect size: 0.2<Cohen’s d≤0.5; large effect size: Cohen’s d≥0.8

| Comparison | Timepoint | Areas of differences (%) | Maximum difference (BW) | Cohen’s d  effect size |
| --- | --- | --- | --- | --- |
| Uninvolved-ACLR | preop | 6-24 | 0.12 | 1.16 |
|  | 2 months | 5-27  74-90 | 0.16  0.09 | 1.53  1.24 |
|  | 4 months | 8-25  77-89 | 0.09  0.06 | 0.82  1.01 |
|  | 6 months | 16-23 | 0.06 | 0.60 |
|  | 12 months | - | - | - |
|  |  |  |  |  |
| Control-ACLR | preop | 10-21  78-84 | 0.06  0.06 | 1.39  1.25 |
|  | 2 months | 12-27 | 0.10 | 1.93 |
|  | 4 months | 17-24 | 0.06 | 1.27 |
|  | 6 months | - | - | - |
|  | 12 months | - | - | - |
|  |  |  |  |  |
| Control-uninvolved | preop | 13-19 | -0.06 | -0.17 |
|  | 2 months | 7-19 | -0.08 | 0.04 |
|  | 4 months | - | - | - |
|  | 6 months | - | - | - |
|  | 12 months | - | - | - |
| ACLR – anterior cruciate ligament reconstruction, BW – body weight, preop – preoperative, vGRF – vertical ground reaction force | | | | |

Table S2.2: Output of the waveform analyses for KFA after adjusting for gait speed at preop, 2, 4, 6, and 12 months post-ACLR between the a) ACLR and uninvolved limb, b) ACLR limb and controls, and c) uninvolved limb and controls, including the areas of gait stance with significant differences, the maximum differences that occurred within those areas as well as the mean Cohen’s d effect sizes for significant areas. Small effect size: Cohen’s d≤0.2; medium effect size: 0.2<Cohen’s d≤0.5; large effect size: Cohen’s d≥0.8

| Comparison | Timepoint | Areas of differences (%) | Maximum difference (°) | Cohen’s d effect Size |
| --- | --- | --- | --- | --- |
| Uninvolved-ACLR | preop | 1-93 | -8.58 | -1.71 |
|  | 2 months | 1-6  11-33  43-90  97-100 | -3.16  3.05  -6.01  2.54 | -0.85  0.52  -1.35  0.50 |
|  | 4 months | 1-4  11-37  49-88 | -2.26  2.64  -3.95 | -0.59  0.45  -0.94 |
|  | 6 months | 10-36  50-87 | 2-53  -3.22 | 0.47  -0.83 |
|  | 12 months | 1-5  48-91 | -1.85  -2.92 | -0.51  -0.64 |
|  |  |  |  |  |
| Control-ACLR | preop | 1-98 | -8.79 | -1.80 |
|  | 2 months | 6-38  49-87  97-100 | 4.32  -4.71  2.39 | 1.09  -1.04  0.72 |
|  | 4 months | 5-39  51-89 | 3.55  -3.84 | 0.87  -0.83 |
|  | 6 months | 1-45  59-82 | 4.84  -2.16 | 0.94  -0.63 |
|  | 12 months | 2-42  58-93 | 2.94  -2.37 | 0.69  .0.61 |
|  |  |  |  |  |
| Control-uninvolved | preop | 92-100 | -1.59 | -0.18 |
|  | 2 months | 1-16  31-50 | 3.17  1.47 | 0.84  0.51 |
|  | 4 months | 1-12 | 2.55 | 0.71 |
|  | 6 months | 1-59 | 3.30 | 0.73 |
|  | 12 months | 1-53 | 2.83 | 0.69 |
| ACLR – anterior cruciate ligament reconstruction, preop – preoperative, KFA – knee flexion angle | | | | |

Table S2.3: Output of the waveform analyses for KEM after adjusting for gait speed at preop, 2, 4, 6, and 12 months post-ACLR between the a) ACLR and uninvolved limb, b) ACLR limb and controls, and c) uninvolved limb and controls, including the areas of gait stance with significant differences, the maximum differences that occurred within those areas as well as the mean Cohen’s d effect sizes for significant areas. Small effect size: Cohen’s d≤0.2; medium effect size: 0.2<Cohen’s d≤0.5; large effect size: Cohen’s d≥0.8

| Comparison | Timepoint | Areas of differences (%) | Maximum difference (BW*height) | Cohen’s d effect Size |
| --- | --- | --- | --- | --- |
| Uninvolved-ACLR | preop | 10-25  44-85 | 0.01  -0.01 | 0.63  -1.27 |
|  | 2 months | 8-34  52-82 | 0.02  -0.01 | 1.43  -1.15 |
|  | 4 months | 9-37  60-81 | 0.01  -0.01 | 0.98  -0.90 |
|  | 6 months | 9-38  67-74 | 0.01  -0.01 | 1.00  -0.54 |
|  | 12 months | 13-27  70-78 | 0.01  -0.01 | 0.48  -0.51 |
|  |  |  |  |  |
| Control-ACLR | preop | 10-31  53-83 | 0.01  -0.01 | 1.44  -1.24 |
|  | 2 months | 7-42  62-79 | 0.02  -0.07 | 2.43  -0.96 |
|  | 4 months | 7-43  65-79 | 0.02  -0.01 | 1.80  -0.69 |
|  | 6 months | 6-46 | 0.02 | 1.78 |
|  | 12 months | 9-40  73-76 | 0.01  -0.01 | 1.20  -0.60 |
|  |  |  |  |  |
| Control-uninvolved | preop | - | - | - |
|  | 2 months | 24-48 | 0.01 | 1.02 |
|  | 4 months | - | - | - |
|  | 6 months | 14-36 | 0.01 | 0.67 |
|  | 12 months | 15-37 | 0.01 | 0.66 |
| ACLR – anterior cruciate ligament reconstruction, BW – body weight, preop – preoperative, KEM – knee extension moment | | | | |

Table S2.4: Output of the waveform analyses for KAM after adjusting for gait speed at preop, 2, 4, 6, and 12 months post-ACLR between the a) ACLR and uninvolved limb, b) ACLR limb and controls, and c) uninvolved limb and controls, including the areas of gait stance with significant differences, the maximum differences that occurred within those areas as well as the mean Cohen’s d effect sizes for significant areas. Small effect size: Cohen’s d≤0.2; medium effect size: 0.2<Cohen’s d≤0.5; large effect size: Cohen’s d≥0.8

| Comparison | Timepoint | Areas of differences (%) | Maximum difference (BW*height) | Cohen’s d effect Size |
| --- | --- | --- | --- | --- |
| Uninvolved-ACLR | preop | - | - | - |
|  | 2 months | 12-23  67-81 | 0.01  0.01 | 0.79  0.45 |
|  | 4 months | - | - | - |
|  | 6 months | - | - | - |
|  | 12 months | - | - | - |
|  |  |  |  |  |
| Control-ACLR | preop | - | - | - |
|  | 2 months | 13-21 | 0.01 | 1.15 |
|  | 4 months | - | - | - |
|  | 6 months | - | - | - |
|  | 12 months | - | - | - |
|  |  |  |  |  |
| Control-uninvolved | preop | - | - | - |
|  | 2 months | - | - | - |
|  | 4 months | - | - | - |
|  | 6 months | - | - | - |
|  | 12 months | - | - | - |
| ACLR – anterior cruciate ligament reconstruction, BW – body weight, preop – preoperative, KAM – knee adduction moment | | | | |
